# PDGF-BB inhibits SP1/Angptl7 mediated chondro-endothelial crosstalk via stress-sensitivity Piezo1 regulation in osteoarthritis

**DOI:** 10.3389/fphar.2026.1853487

**Published:** 2026-07-15

**Authors:** Zhengchao Wang, Jiangxia Cheng, Hongmei Li, Qingsong Zhang, Pengfei Zhu, Yu Cai

**Affiliations:** 1 Department of Sports Medicine, Wuhan Fourth Hospital, Wuhan, China; 2 Hubei Provincial Sports Medicine Center, Wuhan, China; 3 Hubei Key Laboratory of Sports Injury and Precision Treatment, Wuhan Fourth Hospital, Wuhan, China; 4 Hubei Provincial Clinical Research Center for Orthopaedics, Wuhan, China; 5 Department of Anesthesiology, Wuhan Fourth Hospital, Wuhan, China; 6 Zibo First Hospital, Zibo Prevention and Treatment Hospital for Occupation Diseases, Zibo, China; 7 Department of Cardiovascular, Wuhan Fourth Hospital, Wuhan, China; 8 Department of Rehabilitation, Wuhan Fourth Hospital, Wuhan, China

**Keywords:** chondrocyte, endothelial cell, organoid, osteoarthritis, platelet-derived growth factor

## Abstract

**Introduction:**

Osteoarthritis (OA) involves cartilage degradation and subchondral bone alterations, yet the mechanisms of chondro-endothelial crosstalk remain unclear. Stress-sensitive Piezo1 and SP1/Angptl7 signaling may play key roles in this process. This research aimed to investigate whether Platelet-derived growth factor (PDGF)-BB regulates chondro-endothelial crosstalk via Piezo1-mediated SP1/Angptl7 inhibition.

**Methods:**

Single- and multiple-cell-component organoids (chondrocytes SW1353 and endothelial HMEC-1) were constructed using high-throughput 3D culture. Organoids were treated with MIA to induce OA-like changes, followed by PDGF-BB with or without Yoda1 (Piezo1 activator) or Angptl7. *In vivo*, OA was induced in rats by intraarticular MIA injection, and PDGF-BB or sodium hyaluronate (SH)-PDGF was administered. Histology and immunofluorescence were used to assess F-actin formation, Piezo1 activation, SP1 phosphorylation, Angptl7 and VEGF/Notch/DLL4 expression.

**Results:**

PDGF-BB inhibited F-actin formation and reduced Piezo1 activation in OA chondrocytes. It suppressed SP1 phosphorylation and Angptl7 expression, downregulating VEGF/Notch/DLL4 signaling and reducing endothelial invasion in organoids. These effects were partially reversed by Yoda1 and fully reversed by Angptl7. *In vivo*, PDGF-BB and SH-PDGF attenuated cartilage degeneration and reduced Piezo1 activation, SP1 phosphorylation, and Angptl7 expression.

**Discussion:**

PDGF-BB alleviates OA by inhibiting SP1/Angptl7-mediated chondro-endothelial crosstalk, partially via stress-sensitive Piezo1 regulation through cytoskeletal remodeling. Multiple-cell-component organoids provide a valuable *in vitro* model for studying cartilage pathophysiology.

## Introduction

Osteoarthritis (OA) is a degenerative disorder characterized by cartilage degradation and subchondral bone alterations, for which no curative clinical treatments currently exist. Previous studies have primarily focused on repairing damaged cartilage, with less attention paid to subchondral bone repair and the stability of the cartilage microenvironment. Hypoxia has been shown to be essential for maintaining cartilage homeostasis (Zhang et al., 2023). As OA progresses, inflammation and abnormal mechanical stress can disrupt the barrier between cartilage and subchondral bone, leading to vascular invasion from the subchondral bone into cartilage and the collapse of the hypoxic microenvironment (Yang et al., 2022). Our recent study published in 2025 demonstrated that synchronous repair of cartilage and subchondral bone helps stabilize the cartilage microenvironment (Wang et al., 2025b). Endothelial cells play a key role in this vascular invasion process. Meanwhile, chondrocytes produce Angptl7, a secretory cytokine that promotes VEGF/Notch/DLL4-mediated chondro-endothelial signaling via SP1 activation, thereby enhancing vascular invasion (Kimura et al., 2023; Liu et al., 2025; Ren et al., 2024; Sun et al., 2022). Although our previous work showed that inhibition of VEGF/Notch/DLL4 reduces vascular invasion into cartilage, the underlying mechanisms require further investigation (Wang et al., 2025b).

In normal differentiated chondrocytes, the cytoskeleton is primarily composed of spherical G-actin in a free-floating state (Parreno et al., 2017). With OA progression, aberrant inflammatory stimulation activates the RhoA/ROCK signaling pathway, promoting the transformation of G-actin to F-actin, which forms a more rigid linear structure (Jiang et al., 2023; Wang et al., 2025a). This transition leads to a stiffer, less resilient cytoskeletal architecture and induces abnormal micro-stress in chondrocytes (Gan et al., 2024; Gao et al., 2022). Such mechanical stress activates Piezo1, a stress-sensitive ion channel, thereby accelerating OA progression (Gan et al., 2024; Xiao, 2024). Our recent study showed that inhibition of RhoA/ROCK reduces F-actin formation (Wang et al., 2025a). Additionally, published studies have found that specific activation of Piezo1 enhances vascular invasion from subchondral bone into cartilage (Brylka et al., 2024; Li et al., 2022). Therefore, we hypothesize that Piezo1 serves as a critical link between microstructural changes in chondrocytes and chondro-endothelial crosstalk, offering new insights into the cartilage–subchondral bone connection.

Platelet-derived growth factor (PDGF)-BB, a key component of platelet-rich plasma, was initially shown to promote vascular regeneration and has since demonstrated therapeutic potential in OA (Cai et al., 2023; Zhu et al., 2021). Although some studies have raised concerns that its pro-angiogenic effects might exacerbate OA (Su et al., 2020) our previous findings indicate that while PDGF-BB enhances angiogenesis in subchondral bone, it also inhibits vascular invasion into cartilage (Wang et al., 2025b). We initially attributed this effect to the inhibition of VEGF/Notch/DLL4-mediated chondro-endothelial crosstalk, though the underlying mechanisms remained unclear (Wang et al., 2025b). Our recent work also showed that PDGF-BB inhibits F-actin formation via the RhoA/ROCK pathway (Wang et al., 2025a). Based on these observations, we propose that PDGF-BB suppresses F-actin formation, reduces abnormal micro-stress in chondrocytes, inhibits Piezo1 activation, and subsequently downregulates Angptl7/SP1-mediated VEGF/Notch/DLL4 chondro-endothelial crosstalk.

Intraarticularly administered PDGF-BB is rapidly cleared by capillaries and lymphatic vessels in the synovium (Deng et al., 2024). As in our previous studies, we used sodium hyaluronate (SH) as a delivery vehicle to prolong PDGF-BB retention in the joint space, thereby reducing injection frequency and extending therapeutic efficacy *in vivo* (Wang et al., 2025a; Wang et al., 2025b). To better model micro-stress in chondrocytes and chondro-endothelial crosstalk, we employed a low-adhesion grid microchambers designed for high-throughput 3D cell cultures system to construct single-cell-component chondrogenic organoids (Wang et al., 2025a). Building on this, we developed multiple-cell-component organoids containing both chondrocytes and endothelial cells to simulate and investigate chondro-endothelial interactions. This study aims to elucidate the role of PDGF-BB in regulating chondro-endothelial crosstalk via cytoskeletal remodeling—from single-cell-component organoids *in vitro* to multiple-cell-component organoids and finally to an *in vivo* animal model in OA.

## Methods

### Cell culture and organoid construction

SW1353 cells were cultured in high-glucose Dulbecco’s Modified Eagle Medium (DMEM) supplemented with 10% fetal bovine serum and 1% penicillin–streptomycin at a density of 1 × 10^5^ cells/mL (1 × 10^5^ cells/well). HMEC-1 cells were cultured in endothelial cell-specific medium (Procell, Wuhan, China) with 10% fetal bovine serum and 1% penicillin–streptomycin at the same density. All cells were maintained in a humidified atmosphere of 5% CO_2_ at 37° C. The culture medium was refreshed every 2–3 days, and cells were passaged using 0.25% trypsin–EDTA upon reaching 80%–90% confluence.

For organoid construction, SW1353 cells were digested with 0.25% trypsin–EDTA solution to obtain a cell suspension (1 × 10^6^ cells/mL). The low-adhesion grid microchambers (CSwell 600; Jiyan Biology, Suzhou, China), designed for high-throughput 3D cell cultures, were pretreated according to the manufacturer’s instructions. One milliliter of SW1353 cell suspension was added per well to construct chondrocyte organoids comprising 1,000–2,000 cells. After 24 h incubation, the single-cell organoids were constructed completed. Based on the single-cell organoids, HMEC-1 cells were digested with 0.25% trypsin–EDTA solution to obtain a cell suspension (1 × 10^6^ cells/mL) and added to the same chamber. After 24 h incubation, a shell constructed by HMEC-1 cells could be observed outside the SW1353 core and the multiple-cell organoids were constructed completed. Organoid development was confirmed by observation using an inverted microscope (Leica).

### Induction of monosodium iodoacetate (MIA)-induced inflammation and PDGF-BB processing

Recombinant rat PDGF-BB was purchased from R&D Systems, reconstituted, and stored per the manufacturer’s instructions. Monosodium iodoacetate (MIA; Aladdin) was dissolved in normal saline and stored accordingly. Organoid-containing wells were pretreated with PDGF-BB (0 or 100 ng/mL), with or without 5 μM Yoda1 (Piezo1 activator) or 10 ng/mL Angptl7, for 1 h prior to stimulation with 5 μM MIA. Organoids were then incubated for 24 h. Subsequently, organoids were embedded using SE-GEL (Jiyan Biology) following the manufacturer’s protocol and processed for paraffin embedding and histological analysis. The concentration of PDGF-BB (0 or 100 ng/mL) were based on our pervious study (Zhu et al., 2021).

### Preparation of SH-PDGF.

Recombinant rat PDGF-BB was reconstituted to 10 μg/mL and mixed with sodium hyaluronate (SH; Bausch & Lomb, Inc., molecular weight 700,000–1,400,000) at a 1:100 dilution to achieve a final PDGF-BB concentration of 100 ng/mL in SH-PDGF.

### Animals

Male Sprague–Dawley rats (160–200 g) were obtained from the Experimental Animal Center, Tongji Medical College, Huazhong University of Science and Technology. Animals were housed under a 12-h light/dark cycle with controlled humidity and temperature (25 °C) and provided a standard diet. OA was induced in the left knee joint via intraarticular injection of 3 mg/50 μL MIA under 1% pentobarbital anesthesia (80 mg/kg i. p.) using a 27-gauge needle inserted through the patellar tendon. Two weeks post-injection, X-ray imaging was performed using a Faxitron MX-20 system (Faxitron X-ray Corp., Wheeling, IL) to assess joint structure changes. After euthanasia (1% pentobarbital, 80 mg/kg i. p.), radiographs were obtained in the dorsoplantar position at full extension and analyzed by a blinded radiologist. Subsequently, saline (control), PDGF-BB (100 ng/mL), or SH-PDGF (100 ng/mL) was injected into the left knee joint. Seven days after the final injection, all animals were euthanized with an overdose of 10% urethane, and knee joints were collected for histopathological analysis. All procedures complied with the National Institutes of Health Guide for the Care and Use of Laboratory Animals and were approved by the Ethics Committee of Wuhan Fourth Hospital (No. WAEF-2024–0,209). Assessments were performed at week 6 post-OA induction for all groups except the OA (3-week) group. All animals were randomly assigned to each group and the investigators were blinded during the treatment administration and outcome assessment. Each group included five rats, consistent with our previously published study (Wang et al., 2025a), minimizing animal use in accordance with the Declaration of Helsinki.

### Histological analysis

Embedded organoids were fixed in 4% paraformaldehyde for 24 h. Rat knee joint samples were fixed in 4% paraformaldehyde for 24 h and decalcified in 10% EDTA for 4 weeks. Following paraffin embedding, 4-μm-thick sections were prepared for hematoxylin and eosin (H&E) and toluidine blue staining.

### Immunofluorescence (IF) analysis

Histological sections were processed as described. Primary antibodies were diluted according to the manufacturers’ instructions and included: collagen II (1:500; rabbit; Proteintech), CD31 (1:500; mouse; Proteintech), Piezo1 (1:500; rabbit; Proteintech), Angptl7 (1:500; rabbit; Proteintech), SP1 (1:1,000; rabbit; Proteintech), P-SP1 (1:200; rabbit; Proteintech), F-actin (phalloidin; 1:200; Proteintech), VEGF (1:300; rabbit; Proteintech), Notch1 (1:200; rabbit; Proteintech), and DLL4 (1:200; mouse; Proteintech). After deparaffinization and rehydration, sections were incubated with primary antibodies overnight at 4 °C, followed by fluorescein-labeled secondary antibodies for 1 h at room temperature. Nuclei were counterstained with DAPI. Slides were imaged using an LSM 710 confocal microscope (Zeiss, Oberkochen, Germany) with an EC-Plan-Neofluar 40×/1.3 oil immersion objective. Fluorescence intensity was quantified using ZEN 2009 software (Zeiss).

### Statistical analyses

Statistical analyses were performed using IBM SPSS Statistics for Windows, version 21.0 (IBM Corp., Armonk, NY, United States). Normality and was assessed using Q–Q plots. Data were analyzed using ANOVA followed by *post hoc* tests with Bonferroni correction. A p-value <0.05 was considered statistically significant.

## Result

### PDGF-BB modifies histological features and inhibits F-Actin formation in single- and multiple-cell-component organoids in vitro

**FIGURE 1:**
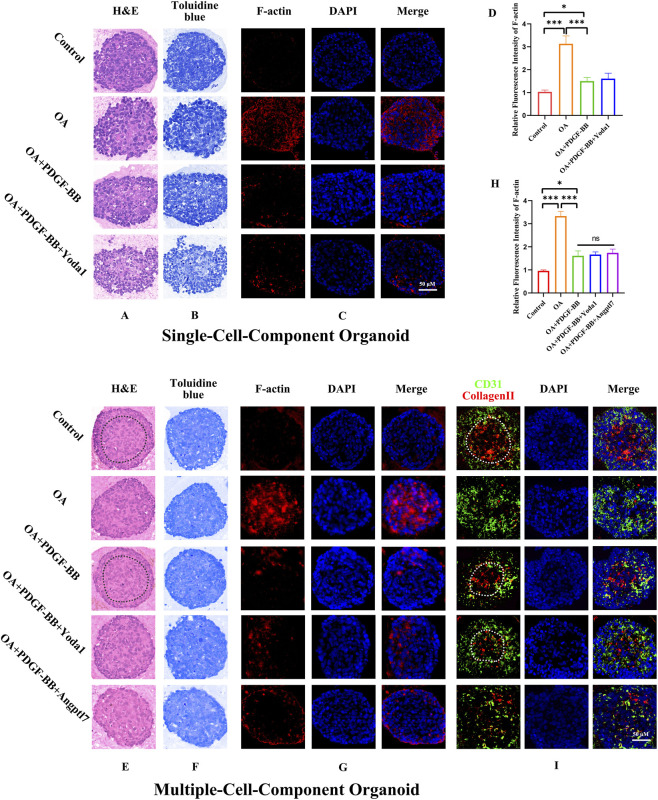
**(A,B)** Formation of single-cell-component organoids assessed by H&E and toluidine blue staining. **(C,D)** Effects of PDGF-BB on F-actin in single-cell-component organoids by immunofluorescence. **(E,F)** Formation of multiple-cell-component organoids assessed by H&E and toluidine blue staining. **(G,H)** Effects of PDGF-BB on F-actin in multiple-cell-component organoids by immunofluorescence. **(I)** Formation of multiple-cell-component organoids assessed by CD31 and collagen II immunofluorescence. N = 3 in all groups. The statistical graph is plotted based on the relative fluorescence intensity. *p < 0.05; ***p < 0.001.

Histological features of organoids were evaluated using H&E staining, toluidine blue staining, and immunofluorescence for CD31/collagen II and F-actin. As shown in Figures 1A,B, no significant histological differences were observed in single-cell-component organoids before or after OA induction, with or without PDGF-BB treatment. OA induction significantly increased F-actin formation, which was attenuated by PDGF-BB (Figures 1C,D). The Piezo1 activator Yoda1 did not affect this response (Figures 1C,D). In multiple-cell-component organoids, a distinct boundary between chondrocyte and endothelial regions was visible by H&E staining and CD31/collagen II immunofluorescence (Figures 1E,I). OA induction disrupted this organization, and PDGF-BB partially restored it (Figures 1E,I). Yoda1 slightly counteracted this effect, while Angptl7 completely reversed it (Figures 1E,I). No significant differences were observed among groups by toluidine blue staining (Figure 1F). OA induction also increased F-actin formation in multiple-cell-component organoids, which was suppressed by PDGF-BB (Figures 1G,H); neither Yoda1 nor Angptl7 influenced this effect (Figures 1G,H). These results suggest that PDGF-BB inhibits F-actin formation and endothelial invasion in OA, with Piezo1 and Angptl7 playing roles in endothelial processes but not in F-actin regulation.

### PDGF-BB Modulates Piezo1 Activation and the SP1/Angptl7 Pathway in Organoids.

**FIGURE 2:**
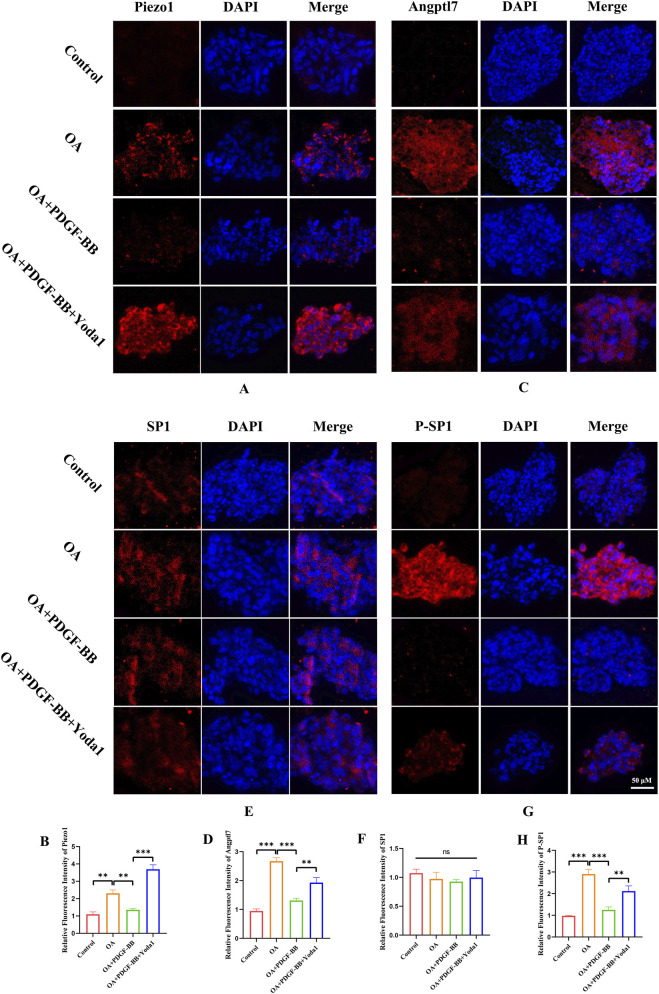
**(A,B)** Effects of PDGF-BB on Piezo1 activation in single-cell-component organoids by immunofluorescence. **(C,D)** Effects of PDGF-BB on Angptl7 expression in single-cell-component organoids by immunofluorescence. **(E–H)** Effects of PDGF-BB on SP1 phosphorylation in single-cell-component organoids by immunofluorescence. ns, not significant; N = 3 in all groups. The statistical graph is plotted based on the relative fluorescence intensity. **p < 0.01; ***p < 0.001.

**FIGURE 3:**
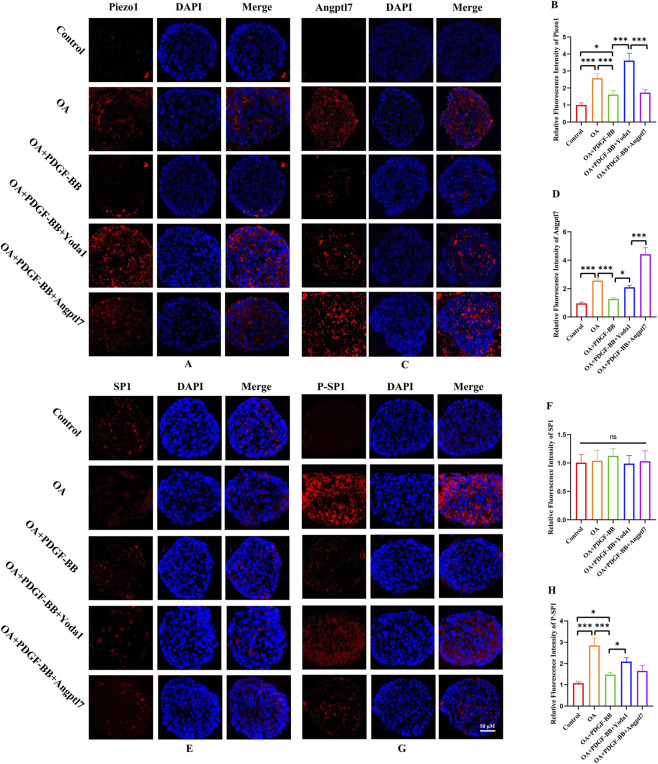
**(A,B)** Effects of PDGF-BB on Piezo1 activation in multiple-cell-component organoids by immunofluorescence. **(C,D)** Effects of PDGF-BB on Angptl7 expression in multiple-cell-component organoids by immunofluorescence. **(E–H)** Effects of PDGF-BB on SP1 phosphorylation in multiple-cell-component organoids by immunofluorescence. ns, not significant; N = 3 in all groups. The statistical graph is plotted based on the relative fluorescence intensity. *p < 0.05; ***p < 0.001.

Immunofluorescence analysis showed that OA induction promoted Piezo1 activation, which was reduced by PDGF-BB (Figures 2A,B, 3A–B). Angptl7 did not alter this effect (Figures 3A,B). OA also enhanced SP1 phosphorylation and Angptl7 expression, both of which were suppressed by PDGF-BB (Figures 2C–H, 3C–H). Yoda1 partially reversed the effect of PDGF-BB on SP1 phosphorylation, whereas Angptl7 had no additional effect (Figures 2C–H, 3–H). These findings indicate that PDGF-BB inhibits the SP1/Angptl7 pathway, partially through Piezo1 regulation.

### PDGF-BB suppresses VEGF/Notch1/DLL4-mediated chondro-endothelial crosstalk in multiple-cell-component organoids

**FIGURE 4:**
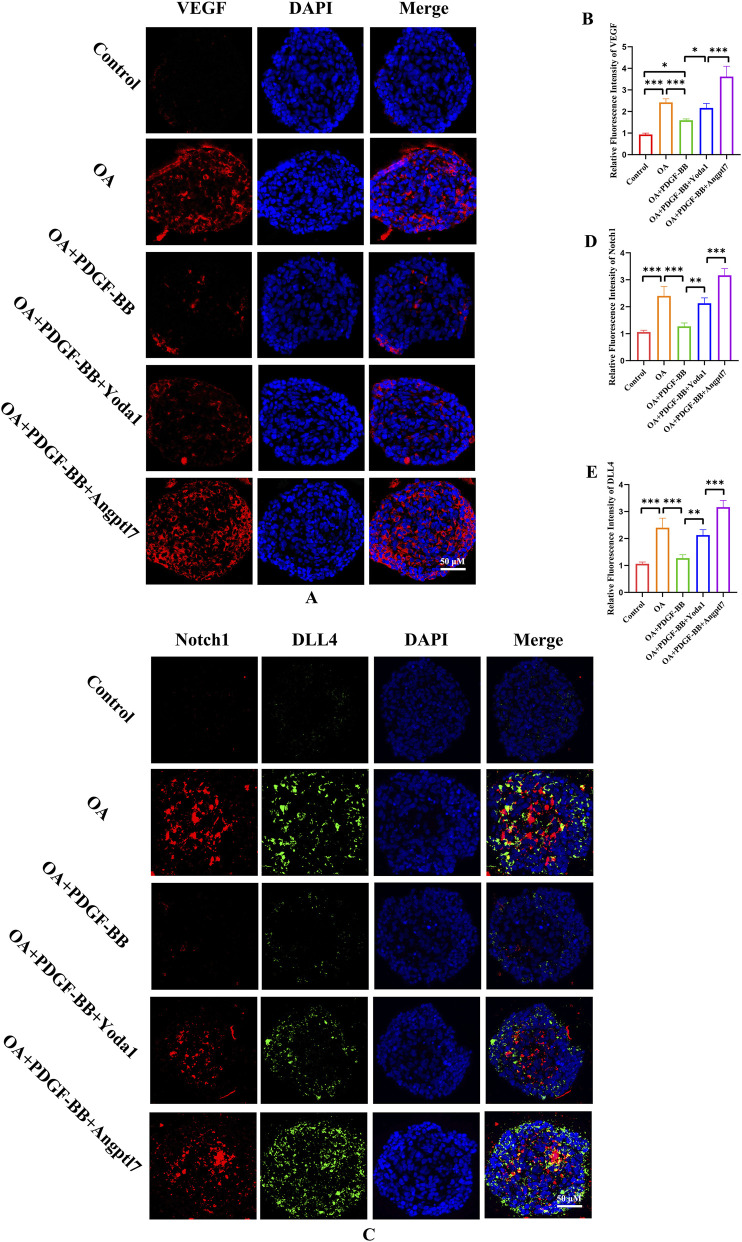
**(A,B)** Effects of PDGF-BB on VEGF expression in multiple-cell-component organoids by immunofluorescence. **(C–E)** Effects of PDGF-BB on Notch1 and DLL4 expression in multiple-cell-component organoids by immunofluorescence. N = 3 in all groups. The statistical graph is plotted based on the relative fluorescence intensity. *p < 0.05; **p < 0.01; ***p < 0.001.

Immunofluorescence revealed that OA induction upregulated VEGF, Notch1, and DLL4 expression, indicating enhanced chondro-endothelial crosstalk (Figure 4). PDGF-BB treatment reduced this upregulation (Figure 4). Yoda1 partially reversed the inhibitory effect of PDGF-BB, while Angptl7 completely abolished it, leading to expression levels even higher than those in the OA group (Figure 4). These results suggest that PDGF-BB inhibits VEGF/Notch1/DLL4 signaling via suppression of Angptl7.

### PDGF-BB and SH-PDGF reduce Piezo1 activation and SP1/Angptl7 signaling in vivo

**FIGURE 5:**
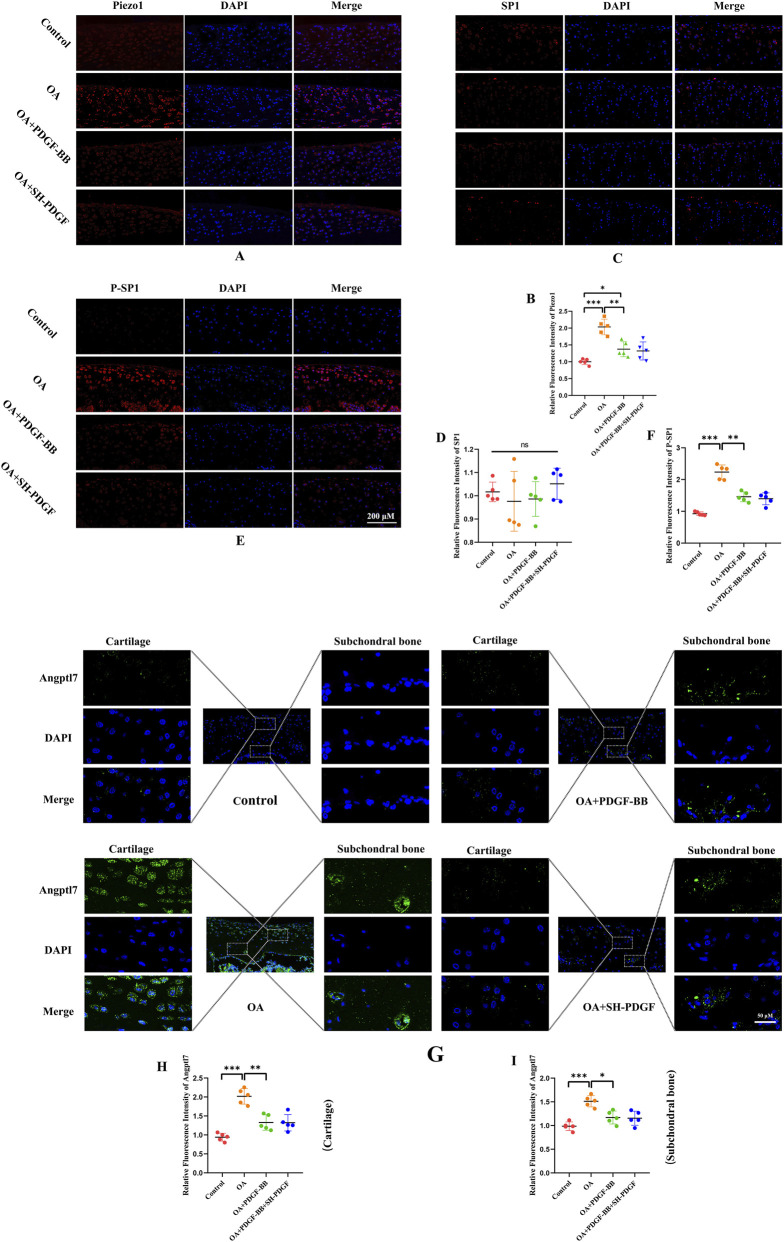
**(A,B)** Effects of PDGF-BB and SH-PDGF on Piezo1 activation in cartilage *in vivo* by immunofluorescence. **(C–F)** Effects of PDGF-BB and SH-PDGF on SP1 phosphorylation in cartilage *in vivo* by immunofluorescence. **(G–I)** Effects of PDGF-BB and SH-PDGF on Angptl7 expression in cartilage and subchondral bone *in vivo* by immunofluorescence. ns, not significant; N = 3 in all groups. The statistical graph is plotted based on the relative fluorescence intensity. *p < 0.05; **p < 0.01; ***p < 0.001.

Our previous studies demonstrated that PDGF-BB and SH-PDGF alleviate cartilage degeneration by modulating VEGF/Notch1/DLL4 signaling between cartilage and subchondral bone in OA (Wang et al., 2025a; Wang et al., 2025b). Here, we further validated the involvement of Piezo1 and the SP1/Angptl7 pathway *in vivo*. OA induction increased Piezo1 activation and SP1 phosphorylation in cartilage, both of which were attenuated by PDGF-BB and SH-PDGF (Figures 5A–F). OA also elevated Angptl7 levels in cartilage and subchondral bone, an effect reversed by PDGF-BB and SH-PDGF (Figures 5G–I). These *in vivo* findings confirm that PDGF-BB inhibits SP1/Angptl7-mediated chondro-endothelial crosstalk via stress-sensitive Piezo1 regulation.

## Discussion

This study presents several key findings. First, PDGF-BB inhibits VEGF/Notch/DLL4-mediated chondro-endothelial crosstalk by downregulating the SP1/Angptl7 signaling pathway in OA. Second, this effect is partially mediated by stress-sensitive Piezo1 inhibition through regulation of F-actin formation in OA chondrocytes. Third, multiple-cell-component organoids constructed using low-adhesion grid microchambers effectively simulate micro-stress and chondro-endothelial interactions, offering a novel *in vitro* platform for cartilage research.

Hypoxia is critical for cartilage metabolism, morphogenesis, and survival (Hu et al., 2020; Sanz-Ramos et al., 2013; Taheem et al., 2020). Our previous work showed that inflammatory stimuli activate the HIF-1α/VEGF/Notch axis, promoting vascular invasion from subchondral bone into cartilage and disrupting the hypoxic microenvironment (Li et al., 2019; Tirpe et al., 2019). Although PDGF promotes chondrocyte proliferation, its pro-angiogenic effects have raised concerns regarding its therapeutic use in OA. While *in vitro* studies using monolayer chondrocyte cultures have suggested that PDGF-BB inhibits OA progression, its *in vivo* effects remain debated. Our earlier investigations addressed this by evaluating PDGF-BB in both cartilage and subchondral bone (Wang et al., 2025b). We found that PDGF-BB promotes angiogenesis in subchondral bone while simultaneously inhibiting vascular invasion into cartilage, an effect partially mediated by RANKL-induced osteoclast formation and cartilage–subchondral bone crosstalk (Wang et al., 2025b). The present study refines this understanding by focusing on chondro-endothelial interactions, demonstrating that VEGF/Notch/DLL4 regulation occurs via the SP1/Angptl7 pathway. These findings underscore that angiogenesis and vascular invasion are not synonymous, and future studies should prioritize chondro-endothelial crosstalk over general angiogenesis.

The transition from G-actin to F-actin is closely linked to chondrocyte microstructural function and differentiation (Parreno et al., 2017). F-actin’s rigid, linear structure induces abnormal micro-stress in chondrocytes (Firat-Karalar and Welch, 2011). Piezo1, a stress-sensitive ion channel, is activated by such mechanical changes. In OA, inflammation promotes F-actin formation, leading to Piezo1 activation, which in turn triggers stress-sensitive signaling cascades and perpetuates a vicious cycle (Brylka et al., 2024; Xiao, 2024). Our findings indicate that Piezo1 activation upregulates the SP1/Angptl7 pathway, enhancing VEGF/Notch/DLL4-mediated chondro-endothelial crosstalk—thereby linking cytoskeletal changes in chondrocytes to endothelial signaling [15]. Our previous study has found that PDGF-BB could inhibit F-actin formation via RhoA/ROCK inhibition in OA (Wang et al., 2025a). The current study extends this by showing that inhibition of F-actin formation downregulates Piezo1 activation and that PDGF-BB suppresses SP1/Angptl7 signaling partially through Piezo1 inhibition. These results highlight the interconnectedness of chondrocyte cytoskeletal dynamics, micro-stress, and chondro-endothelial crosstalk, emphasizing the need to consider these elements as an integrated system in OA pathophysiology.

Most previous cartilage organoid models have relied on stem cells such as embryonic stem cells (ESCs), induced pluripotent stem cells (iPSCs), mesenchymal stem cells (MSCs), adult stem cells (ASCs) and human periosteum-derived cells (hPDCs) (Abe et al., 2023; Abraham et al., 2022; Jiang et al., 2021; Lin et al., 2023). While useful for disease modeling and tissue engineering, these systems are limited by complex culture requirements and unpredictable differentiation. As noted, chondrocyte microstructure, micro-stress, crosstalk, and microenvironment form a unified system that monolayer cultures cannot replicate. In our prior work, we demonstrated that tumor-derived cell lines can be used to construct cartilage organoids that model micro-stress and are suitable for high-throughput applications (Wang et al., 2025a). Here, we extended this approach by developing multiple-cell-component organoids incorporating both chondrocytes and endothelial cells to simulate chondro-endothelial crosstalk. Organoids generated using low-adhesion grid microchambers (CSWell 600) offer a practical, reproducible, and scalable platform for *in vitro* cartilage research. Their ease of construction, controllable growth, and proliferative capacity make them well-suited for disease modeling and drug screening. However, this represents an initial step; further optimization and validation are needed to fully realize their potential.

Our previous studies have found the anti-vessel-invasion effect and micro-stress-improvement effect of PDGF-BB in OA (Wang et al., 2025a; Wang et al., 2025b). In this study, based on multiple-component-organoids, a connection was established between the two biological processes Piezo1 and SP1/Angptl7 axis. Still, in the context of exploring novel OA novel treatments, single chondrocytes viability improvement is only a part of the mechanism. The chondrocytes, inter-chondrocyte micro-stress, and the microenvironment around chondrocytes is an integrated organic whole, which could provide novel insights. In addition, PDGF-BB is known to exert multiple biological effects. Our group has paid much attention to the mechanisms of PDGF-BB in OA since 2021 and various mechanisms and biological effects were reported (Cai et al., 2023; Zhu et al., 2021). We must admit that the results presented in this research may only be the tip of the iceberg. Indeed, additional mechano-transduction or angiogenic pathways may also contribute to the observed responses, which may connect the multiple functions into a rational network. This will be the goal of future investigations.

This study has several limitations. First, the use of SW1353 and HMEC-1—both tumor-derived cell lines—may not fully recapitulate the behavior of primary chondrocytes and endothelial cells *in vivo*. They may exhibit certain transformed phenotypes. As results, this strategy could be only applied for phenotypic simulation. The further application needs to be explored in future. Second, while SH was used as a sustained-release carrier for PDGF-BB based on previous work, its clinical applicability requires further validation. Third, we performed with three biological replicates based on our previous published study (Wang et al., 2025a). However, there is no universally accepted standard has been established of the detection replicates based on organoids. The statistical standards of studies with organoids need to be further explored.

## Conclusion

**FIGURE 6:**
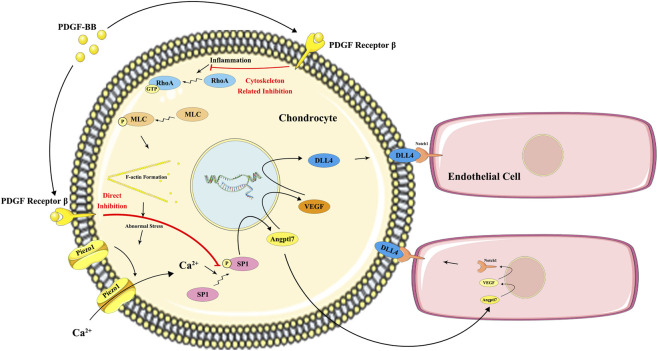
Summary schematic. PDGF-BB alleviates OA by inhibiting SP1/Angptl7-mediated VEGF/Notch/DLL4 chondro-endothelial crosstalk, partially through stress-sensitive Piezo1 inhibition via regulation of F-actin formation.

In summary as shown in Figure 6, this study demonstrates that PDGF-BB alleviates OA by inhibiting SP1/Angptl7-mediated VEGF/Notch/DLL4 chondro-endothelial crosstalk, an effect partially mediated by stress-sensitive Piezo1 inhibition through regulation of F-actin formation.

## Data Availability

The raw data supporting the conclusions of this article will be made available by the authors, without undue reservation.
